# Advances in genomics of bony fish

**DOI:** 10.1093/bfgp/elt046

**Published:** 2013-11-29

**Authors:** Herman P. Spaink, Hans J. Jansen, Ron P. Dirks

**Keywords:** fish models, teleosts, genomics, aquaculture, next-generation sequencing, zebrafish, medaka

## Abstract

In this review, we present an overview of the recent advances of genomic technologies applied to studies of fish species belonging to the superclass of Osteichthyes (bony fish) with a major emphasis on the infraclass of Teleostei, also called teleosts. This superclass that represents more than 50% of all known vertebrate species has gained considerable attention from genome researchers in the last decade. We discuss many examples that demonstrate that this highly deserved attention is currently leading to new opportunities for answering important biological questions on gene function and evolutionary processes. In addition to giving an overview of the technologies that have been applied for studying various fish species we put the recent advances in genome research on the model species zebrafish and medaka in the context of its impact for studies of all fish of the superclass of Osteichthyes. We thereby want to illustrate how the combined value of research on model species together with a broad angle perspective on all bony fish species will have a huge impact on research in all fields of fundamental science and will speed up applications in many societally important areas such as the development of new medicines, toxicology test systems, environmental sensing systems and sustainable aquaculture strategies.

## INTRODUCTION

In the recent years there have been tremendous advances in genomic studies of many vertebrate species. In these studies the attention to various representatives of the bony fish species (the superclass of Osteichthyes) has been increasing enormously, especially focussing on the infraclass of Teleostei that represent approximately 96% of the species of this superclass. This increase in attention is partly the result of the fact that this superclass with about 27 000 living species represents more than 50% of all known vertebrate species [1–4]. In our opinion, it also reflects the trend that fundamental and applied scientific interests in the genomics of bony fish are now converging. On the one hand, fish species such as zebrafish and medaka have clearly shown their broad applicability for studies of fundamental processes underlying development and disease. The tremendous attention these fish species have obtained for an extensive range of fundamental and applied research purposes have earned them the qualification of model fish species. On the other hand, the economical value of the bony fish for food resources coincides with their applicability for biomedical applications and toxicology studies. Together, these fundamental and applied scientific purposes have made it possible that the most advanced genomics technologies have been used for studies of many bony fish species, ranging from the model fish species zebrafish and medaka to ‘living fossils’ such as the coelacanths and the fresh water eels [5–11]. The fresh water eels have only recently been termed living fossils since apparently they have retained most of the genome duplication that occurred after the radiation of the bony fish from the common ancestor with the mammals. This is an example that these studies already are giving an unprecedented insight into the evolution of all bony fish species. The teleost species are extremely interesting for evolutionary studies because they are widespread in an incredible range of microenvironments containing water, ranging from the deepest levels of the oceans, to caves completely devoid of any light or even in environments which most of a year do not contain any water. This has led to remarkable adaptations to life at extreme conditions as exemplified by the tilapia species that can survive at 44°C at very high salinity, Antarctic toothfish that can thrive at temperatures below 0°C and deep sea fish such as from the genus *Coryphaenoides* that can stand pressures of more than 60 MPa [2, 12]. This has made bony fish species very attractive for studies on the effects of adverse conditions such as high gravity that are applicable to space travel research [13–15], or the absence of light that has important implications for studies of circadian rhythm in adults and embryonic stages [16–20]. On the other hand, the response of many bony fish species such as trouts and minnows to toxic compounds is very similar to that in humans. Therefore, these fish have been extensively used for toxicology research already for many decades [21–24] and recently this attention has been extended to the model fish species zebrafish and medaka [25–32]. In this review, we will give an overview of genome sequencing and assembly technologies that have been most popular to study the bony fish and the near future possibilities that will still have to gain in importance. Secondly, we will discuss the impact of fundamental and applied research on model fish species with special attention to the current status of genome sequencing and the impact for further genomic studies. Thirdly, we will give an overview of the advances in genomics of non-model bony fish species. Finally, we will discuss the predicted impact of bony fish genomics on biomedical and aquacultural applications and their importance for future evolutionary studies in a broader perspective than the bony fish.

## COMPARISON OF SEQUENCING PLATFORMS

Over the past 8 years a number of so-called next-generation sequencing platforms have hit the market. They are all based on parallel sequencing of immobilized targets and have revolutionized the genomics field by generating an abundance of sequencing data. Several different sequencing strategies are employed by these platforms. Each of them has their own characteristics. Here we will briefly discuss some of the more popular platforms which are widely used in fish genomics today. An overview of several characteristics of these platforms is shown in Table 1.

**Table 1:** Overview of high-throughput sequencing platforms

Platform	Roche 454 FLX +	Life Technologies SOLiD 5500XL	Illumina HiSeq High Output	Illumina HiSeq Rapid Run	Illumina MiSeq	Pacific Biosciences PacBio RS II	Life Technologies Ion Torrent PGM	Life Technologies Ion Torrent Proton
Mean read length (bp)	700	2 × 60	2 × 100	2 × 150	2 × 250	4500	400	170
Reads/run	∼1 M	1.4 G	6 G	1.2 G	30 M	40–60 K	∼5 M	60–80 M
Yield/run	0.7 Gb	155 Gb	600 Gb	120 Gb	8 Gb	230 Mb	1 Gb	8–10 Gb
Raw error rate	<1%	∼5%	∼0.1%	∼0.1%	∼0.1%	∼15%	0.5–2%	<1%
Run time	23 h	8 days	11 days	27 h	39 h	120 min	7 h	4 h
Technology	Pyrosequencing with luciferase detection.	Ligation system with fluorescent dibase tags.	Single nucleotides are incorporated into the synthesized strand, imaged. The terminator is removed after imaging allowing incorporation of the next nucleotide.			Live imaging of fluorescent strand synthesis.	Pyrosequencing with pH detection.	
Remarks	Short runtime.	Short read length.	Lower coverage on AT- and GC-rich sequences.			Long read length.	Short runtime.	
			Errors accumulate at end of read.			No sequence bias.	Homopolymers cannot be properly resolved.	
	Homopolymers cannot be properly resolved.	Low coverage on GC-rich sequences.	Short run time in Rapid run and on MiSeq.			High raw error rate.	Low coverage on AT-rich sequences.	
Cost/Mb	$10.00	$0.07	$0.05	$0.05	$0.14	$3.00	$1.00	$0.10

There are now four companies who together dominate the market. Roche (454 GS FLX) and Life Technologies (Ion Torrent machines) both developed systems that use pyrosequencing to read the DNA sequence. Although this technique is fast it has problems reading through homopolymers. The read length on the Ion Torrent machine does not match these from the 454 GS FLX but is likely to increase as new chips and chemistry become available.

Next to their Ion Torrent machines Life Technologies also has the SOLiD platform in its portfolio. This platform is more comparable in terms of throughput and costs per base to the Illumina platform. Whereas SOLiD employs a ligation system with dibase tags, Illumina’s HiSeq and MiSeq use a process called sequencing by synthesis (SBS). This SBS technology has already been on the market for a few years now and lately the development of this technology has mainly resulted in longer read length and not so much in more reads per flow-cell.

All these machines need clonal copies of the DNA molecule to obtain enough signal for reliable base calling. The amplification step needed to obtain these copies can be a source of bias in the sequence data and information about DNA modifications is lost.

An altogether different system is used by the PacBio RS II from Pacific Biosciences. In this machine strand synthesis is followed on single DNA molecules. Although this produces reads spanning several kilobases the raw error rate is high due to the nature of imaging single molecules. Since no amplification is needed it has the benefit that DNA modifications can also be detected and there is no bias in the sequence data.

When using different applications like *de novo* genome sequencing, resequencing and transcriptome sequencing different parameters are important that influence the choice of the sequencing platform. For *de novo* genome sequencing it is important to have even coverage in all regions and to have a low error rate. To facilitate assembly the read length should be as long as possible. The combined use of Illumina HiSeq and PacBio RS platforms are best suited for this type of applications. When sequencing a transcriptome a high throughput is desirable but read length is a less important factor.

In the coming years we can expect a further drop in cost/Mb driven by ongoing development of the current technologies and the introduction of new sequencing technologies like sequencing using nanopores. This will result in tools that will make *de novo* genome sequencing and resequencing even more efficient and easier.

The sequencing endeavours of non-model fish species are increasingly based on whole genome shotgun sequencing (WGS). This kind of sequence data is still inferior in coverage to map-based sequence data, for instance based on BAC sequencing. This is notwithstanding the fact that even in the absence of large scaffolded WGS data sets it is still possible to obtain highly valuable complete exome predictions that also make use of transcriptome data sets and improved gene prediction models.

However, especially chromosomal areas with many repetitive sequences will be poorly covered by WGS assemblies. Furthermore, for polyploid species it will be very difficult to obtain a reliable estimate of the coverage of the entire genome. The bioinformatics needed for scaffolding of WGS is still in the development stage. In Table 2, we present an overview of the software that has been used for *de novo* assembly and scaffolding of WGS data. It can be argued that in the future the technologies mentioned above will further improve to such extent that the disadvantages of WGS will become less pronounced. For instance, when PacBio sequencing length runs and coverage will further increase it could be used to obtain larger scaffolds even for difficult areas of a WGS assembly. This was recently demonstrated by sequencing the genome of the Arabidopsis Ler-0 mutant solely using the PacBio RS II platform (data available from github.com/PacificBiosciences/DevNet/wiki/Datasets).

**Table 2:** An overview of software packages that have been used for the *de novo* assembly and scaffolding of fish genomes

Software package	Software Reference	Web resource	Genome assemblies using this software	Genome Reference
Arachne	Jaffe *et al.*, 2003 [33]	ftp://ftp.broadinstitute.org/pub/crd/ARACHNE/	*Petromyzon marinus* (sea lamprey)	Smith *et al.*, 2013 [34]
Bowtie	Langmead *et al.*, 2009 [35]	http://bowtie-bio.sourceforge.net/index.shtml	*Thunnus orientalis* (Pacific bluefin tuna)	Nakamura *et al.*, 2013 [36]
Celera Assembler	Myers *et al.*, 2000 [37]	http://wgs-assembler.sourceforge.net	*Callorhinchus milii* (elephant shark)	Venkatesh *et al.*, 2007 [38]
			*Gadus morhua* (Atlantic cod)	Star *et al.*, 2011 [39]
			*Takifugu rubripes* (pufferfish)	Aparicio *et al.*, 2002 [40]
CLCBio Assembly Cell	N.A.	http://www.clcbio.com/products/clc-assembly-cell/	*Anguilla japonica* (Japanese eel)	Henkel *et al.*, 2012a [6]
			*Cyprinus carpio* (common carp)	Henkel *et al.*, 2012 [41]
CLCBio Genome Workbench	N.A.	http://www.clcbio.com/products/clc-genomics-workbench/	*Leucoraja erinacea* (little skate),	King *et al.*, 2011 [42]
			*Xiphophorus maculatus* (platyfish)	Schartl *et al.*, 2013 [43]
			*Anguilla anguilla* (European eel)	Henkel *et al.*, 2012b [7]
Fuzzypath	Sudbery *et al.*, 2009 [44]	ftp://ftp.sanger.ac.uk/pub/zn1/fuzzypath/	*Danio rerio* Zv9 (zebrafish)	Howe *et al.*, 2013 [9]
Newbler/GS De Novo Assembler	Margulies *et al.*, 2005 [45]	http://www.454.com/products/analysis-software/	*Gadus morhua* (Atlantic cod)	Star *et al.*, 2011 [39]
			*Xiphophorus maculatus* (platyfish)	Schartl *et al.*, 2013 [43]
			*Thunnus orientalis* (Pacific bluefin tuna)	Nakamura *et al.*, 2013 [36]
PCAP	Huang *et al.*, 2003 [46]	http://seq.cs.iastate.edu/pcap.html	*Xiphophorus maculatus* (platyfish)	Schartl *et al.*, 2013 [43]
Phrap	De la Bastide and McCombie, 2007 [47]	http://www.phrap.org/phredphrapconsed.html	*Labeotropheus fuelleborni* (blue mbuna)	Loh *et al.*, 2008 [48]
			*Maylandia zebra* (Zebra Mbuna)	
			*Mchenga conophoros*	
			*Melanochromis auratus* (golden Mbuna)	
			*Rhamphochromis esox*	
Phusion	Mullikin and Ning, 2003 [49]	http://www.sanger.ac.uk/resources/software/phusion/	*Danio rerio* Zv9 (zebrafish)	Howe *et al.*, 2013 [9]
RAMEN Assembler	N.A.	N.A.	*Oryzias latipes* (Medaka)	Kasahara *et al.*, 2007 [50]
				Ahsan *et al.*, 2008 [51]
SSPACE Scaffolder	Boetzer *et al.*, 2011 [52]	http://www.baseclear.com/landingpages/basetools-a-wide- range-of-bioinformatics-solutions/sspace-premium/	*Anguilla anguilla* (European eel)	Henkel *et al.*, 2012 [41]
			*Anguilla japonica* (Japanese eel)	Henkel *et al.*, 2012a [6]
			*Cyprinus carpio* (common carp)	Henkel *et al.*, 2012b [7]

It should also be mentioned that alternative methods to BAC sequencing have been developed that are highly applicable to obtaining genetic maps of fish species. To obtain a genetic map of an organism restriction associated DNA (RAD) tag sequencing can be employed as demonstrated for the spotted gar [53], the threespine stickleback [54] and the *Xiphophorus* sequencing projects [43]. This method uses next-generation sequencing to map sequence variants in the neighbourhood of restriction sites in the offspring from a cross. From the inheritance of the variants a high-density genetic linkage map can be constructed. This map can then be used to align scaffolds in higher order structures. More recently optical mapping of nicking sites on the genome in nanochannel arrays has also been employed to create a high-density genome map that can be used to order contigs and scaffolds [55].

## GENOMICS IN MODEL FISH SPECIES

The most frequently studied fish species are zebrafish (*Danio rerio*) and medaka (*Oryzias latipes*). Although statistically the zebrafish is currently used most often as a research model, the use of medaka has particular advantages and the importance of the availability of two genomically well-characterized models for comparative purposes and tool development should not be underestimated [5, 56, 57]. For instance, the use of the Tol2 transposon from medaka in the zebrafish, where this transposon does not occur, is the basis for the most successful transgenesis protocols in zebrafish [58]. As a result of the combined efforts of a very large number of research groups these fish species have now established themselves in every field of biology, and also have propagated the use of fish species for chemical, physical and mathematical studies [59–61] and therefore have earned the name model fish species. Although historically these models have earned their fame by their contribution to large forward genetic screens linked to vertebrate developmental studies [62], in recent years these model species have also been extensively used for biomedical applications, and there are already several examples of medicines in clinical trials that were originally developed in zebrafish models. These studies have shown that research in model fish species can greatly speed up the discovery of new medicines [63–66]. Model fish species are also increasingly used for comparative studies in experiments with other fish species that are of importance for aquaculture, e.g. as a model for the effects of swimming exercise on muscle development [67]. Reversely, species that are very important in aquaculture, such as rainbow trout and common carp (*Cyprinus carpio*), have shown to have benefits for fundamental research. Research with the latter species is especially relevant to biomedical studies in the very closely related zebrafish owing to its large body size, the availability of highly inbred lines and a very large spawn size that offers possibilities for high-throughput screening [41, 68].

From a genomics perspective the zebrafish genome is now the most advanced model in that the sequencing efforts have reached the stage in which the completed genome will be further perfected by the Genome Reference Consortium (http://genomereference.org) [9]. The recently published zebrafish reference genome will undoubtedly have a major impact on future genomics studies, for instance by its major role in aiding the identification of protein functions, as shown recently by Kettleborough *et al.* [69] and Varshney *et al.* [70], and by supporting the identification of mutations in forward genetic screens [71]. Howe *et al.* [9] have shown examples of how the available genomic sequence data can lead to new insights into the evolution of genome architecture and can identify new biological functions for instance involved in sex determination. The results obtained from the zebrafish models can now be compared with other fish species such as medaka that has been extensively used for studies of sex determinants and is thereby the basis to obtain a better understanding of the evolution of sex determination in all bony fish with implications for mammalian research on sex chromosome evolution [72–75]. Due to the rapid evolutionary turn-over of sex chromosomes in fish, sex-linked markers found in medaka and zebrafish will not be directly translatable to results in other fish species. However, by comparative genomic studies with the data obtained in species such as medaka and rainbow trout [76] the resulting knowledge on sex determination mechanisms in several bony fish might also lead to predicted gender markers for other fish species. This will have applications for aquaculture, since methods for determining the sex ratios of offspring of cultured fish species is of economical value.

The genome sequence of the zebrafish demonstrates that even between closely related fish species there can be large differences in repetitive DNA content. For instance, in zebrafish the type II DNA transposable elements cover 39% of the entire genome sequence [9], whereas in common carp there is a very low number of repetitive elements, as low as in fugu [41]. This, together with smaller intron and intergenic region sizes, explains why common carp as a pseudo-tetraploid species has a similar DNA content as zebrafish. We recently have obtained a shotgun sequence of the giant Danio (genus *Devario*) showing that it has a diploid genome that resembles the zebrafish rather than common carp in its richness of repeat sequences (Spaink and Dirks, unpublished data).

In addition to these comparative studies, the available model fish genome sequences are an essential basis for the successful interpretation of the extensive transcriptome, proteome and metabolome data sets that are now rapidly accumulating, also for non-model fish species, as illustrated by a small representation of the many recent publications that have stimulated our research in this area [41, 77–93]. The limited annotation of particular classes of genes, such as non-coding RNAs and genes that are only expressed during disease, are bottlenecks that still need to be addressed. Furthermore, there is still a lack of information on orthology relationships between genes from different fish species and mammalian genes. This is a pity since the application in model fish of many new genomics technologies, for instance in epigenetic analysis [94–98], will be more difficult to translate to comparative epigenetic studies in other fish species and mammals.

## NEW INSIGHTS FROM NON-MODEL TELEOST FISH GENOMES

Commercial availability of massive parallel sequencing or next-generation sequencing technologies in 2005 triggered an exponential growth of the number of species for which draft assemblies of complete genome sequences were released. The genome sequence of the giant panda was the first sequence of a vertebrate species that was *de novo* assembled based on next-generation technology alone [99]. As of 2 July 2013 a total of 3263 eukaryotic genomes were registered at NCBI's genome database (http://www.ncbi.nlm.nih.gov/genome/). Animal genomes accounted for 977 entries and the majority of these belong to the groups of mammals (378) and insects (285). Teleost fish, although the largest known group of vertebrates (∼ 27 000 species), are only poorly represented in this database, namely by 93 species and including 42 entries with the status ‘no data’ and 17 entries with the status ‘SRA/traces’. A combined search for whole genome sequencing projects of ray-finned fish (Actinopterygii) and lobe-finned fish (Sarcopterygii) in three commonly used databases, namely NCBI, ENSEMBL (http://www.ensembl.org/index.html) and GOLD (www.genomesonline.org/), resulted in a list of 61 registered fish genomics projects (Table 3), some of which have the status ‘Scaffolds or contigs’ (27), or ‘Chromosomes’ (6), and more than half of which are still incomplete. Clearly, the orders of the Cypriniformes (6 projects), Cyprinodontiformes (11 projects) and Perciformes (18 projects) are currently the most popular for genomics projects.

**Table 3:** Whole genome sequencing projects (as of 2 July 2013) of ray-finned fish (Actinopterygii) and ^a^lobe-finned fish (Sarcopterygii)

Organism/name	Order; family	Common name	NCBI Project ID	GoldCARD ID	Size (Mb)	GC%	Chrs	WGS	Scaffolds	Status	Ref.
*Acanthemblemaria maria*	Perciformes; Chaenopsidae	Secretary blenny	PRJNA175737	Gi0044402	–	–	–	–	–	–	–
*Anguilla anguilla*	Anguilliformes; Anguillidae	European eel	PRJNA73577	Gi0045243	–	–	–	–	–	–	[7]
*Anguilla japonica*	Anguilliformes; Anguillidae	Japanese eel	PRJNA158309	Gi0053798	–	–	–	–	–	–	[6]
*Anoplopoma fimbria*	Scorpaeniformes; Anoplopomatidae	Sablefish	PRJNA202249	Gi0048049	–	–	–	–	–	–	–
*Aphanius shirini*	Cyprinodontiformes; Cyprinodontidae	–	PRJNA203365	Gi0049840	–	–	–	–	–	–	–
*Astronotus crassipinnis*	Perciformes; Cichlidae	‘Fat Oscarfish’	PRJNA167777	Gi0044952	–	–	–	–	–	–	–
*Astyanax mexicanus*	Characiformes; Characidae	Mexican tetra	PRJNA89115	Gi0044658	964.31	37.9	–	APWO01	10735	Scaffolds or contigs	–
*Carassius auratus* red var.	Cypriniformes; Cyprinidae	Red crucian carp	PRJNA80997	Gi0045250	–	–	–	–	–	–	–
*Chaenocephalus aceratus*	Perciformes; Channichthyidae	Blackfin icefish	PRJNA89117	Gi0044639	–	–	–	–	–	–	–
*Clarias fuscus CLFUWH01*	Siluriformes; Clariidae	Whitespotted clarias	PRJNA38195	Gi06053	–	–	–	–	–	–	–
*Coilia nasus COECWH01*	Clupeiformes; Engraulidae	Japanese grenadier anchovy	PRJNA38187	Gi06054	–	–	–	–	–	–	–
*Ctenopharyngodon idella*	Cypriniformes; Cyprinidae	Grass carp	PRJNA30857	Gi06056	–	–	–	–	–	–	–
			PRJNA39737	Gi07179							
*Cynoglossus semilaevis*	Pleuronectiformes; Cynoglossidae	Tongue sole	PRJNA73987	Gi0043417	–	–	–	–	–	–	–
*Cyprinodon variegatus*	Cyprinodontiformes; Cyprinodontidae	Sheepshead minnow	PRJNA89149	Gi0044689	–	–	–	–	–	–	–
*Cyprinus carpio carpio*	Cypriniformes; Cyprinidae	Common carp	PRJNA73579	Gi0045244	–	–	–	–	–	–	[41]
*Danio rerio*	Cypriniformes; Cyprinidae	Zebrafish	PRJNA13922	–	1412.47	36.7	25	CABZ01	4560	Chromosomes	[9]
			PRJNA11776	Gc00272							
*Dicentrarchus labrax*	Perciformes; Moronidae	European seabass	PRJEA39865	Gi07181	98.25	40.3	–	CABK01	–	Scaffolds or contigs	–
*Engraulis encrasicolus*	Clupeiformes; Engraulidae	European anchovy	PRJNA202430	Gi0048051							
*Gadus morhua*	Gadiformes; Gadidae	Atlantic cod	PRJNA41391	Gi05656	608.29	45.6	–	CAEA01	427427	Scaffolds or contigs	[39]
*Gasterosteus aculeatus*	Gasterosteiformes; Gasterosteidae	Three-spined stickleback	PRJNA13579	Gi00269	446.62	44.6	–	AANH01	–	Scaffolds or contigs	–
*Haplochromis burtoni (*Astatotilapia burtoni)	Perciformes; Cichlidae	–	PRJNA60363	Gi03070	698.98	40.5	–	AFNZ01	8001	Scaffolds or contigs	–
*Labeotropheus fuelleborni*	Perciformes; Cichlidae	Blue mbuna	PRJNA29479	Gi03371	69.35	42.2	–	ABPK01	58245	Scaffolds or contigs	[48]
*Lateolabrax japonicus*	Perciformes; Lateolabracidae	Japanese sea bass	PRJNA38197	Gi07170	–	–	–	–	–	–	–
*Latimeria chalumnae*^a^	Coelacanthiformes; Latimeriidae	African coelacanth	PRJNA56111	Gi08350	2183.72	41.2 42.0	–	AFYH01	22818	Scaffolds or contigs	[100]
			PRJDB500	–	2612.11		–	BAHO01	–	Scaffolds or contigs	–
*Latimeria menadoensis*^a^	Coelacanthiformes; Latimeriidae	Indonesian coelacanth	PRJNA38001	Gi04473	–	–	–	–	–	–	[101]
*Leiocassis longirostris*	Siluriformes; Bagridae	Chinese longsnout catfish	PRJNA38185	Gi07164	–	–	–	–	–	–	–
*Lepisosteus oculatus*	Lepisosteiformes; Lepisosteidae	Spotted gar	PRJNA68247	Gi0043560	945.86	40.4	29	AHAT01	2105	Chromosomes	–
*Leptobotia elongata*	Cypriniformes; Cobitidae	Royal clown loach	PRJNA205477	Gi0049849	–	–	–	–	–	–	
*Maylandia zebra*	Perciformes; Cichlidae	Zebra mbuna	PRJNA29483	Gi03072	77.03	42.5 28.0	–	ABPM01	65094	Scaffolds or contigs	[48]
			PRJNA198780	–	713.57		–	AGTA02	3725	Scaffolds or contigs	–
*Mchenga conophoros (Copadichromis conophorus)*	Perciformes; Cichlidae	–	PRJNA29477	Gi03370	71.43	41.9	–	ABPJ01	61923	Scaffolds or contigs	[48]
			–	Gi18480	–	–	–	–	–	–	
*Melanochromis auratus*	Perciformes; Cichlidae	Golden mbuna	PRJNA29481	Gi03369	66.55	41.6	–	ABPL01	63297	Scaffolds or contigs	[48]
*Mulloidichthys flavolineatus*	Perciformes; Mullidae	Yellowstripe goatfish	PRJNA184890	Gi0045086	–	–	–	–	–	–	–
*Neolamprologus brichardi*	Perciformes; Cichlidae	Princess of Burundi	PRJNA60365	Gi08440	685.96	40.4	–	AFNY01	9098	Scaffolds or contigs	–
*Nothobranchius furzeri*	Cyprinodontiformes; Nothobranchiidae	Turquoise killifish	PRJNA29535	Gi04460	5.32	44.9 44.3	–	ABLO01	5299	Scaffolds or contigs	[102]
			PRJNA33315	Gi04461	5.25		–	ACCZ01	5617	Scaffolds or contigs	[102]
*Nothobranchius kuhntae*	Cyprinodontiformes; Nothobranchiidae	Beira killifish	PRJNA33401	Gi04462	5.24	44.8	–	ACDA01	5934	Scaffolds or contigs	[102]
*Notothenia coriiceps*	Perciformes; Nototheniidae	Black rockcod	PRJNA66471	Gi0044648	–	–	–	–	–	–	–
*Oncorhynchus mykiss*	Salmoniformes; Salmonidae	Rainbow trout	PRJNA172149	Gi0044272	–	–	–	–	–	–	–
*Opsanus beta*	Batrachoidiformes; Batrachoididae	Gulf toadfish	PRJNA196921	Gi0048061	–	–	–	–	–	–	–
*Oreochromis niloticus*	Perciformes; Cichlidae	Nile tilapia	PRJNA72943	–	816.12	40.4	–	AERX01	5901	Scaffolds or contigs	–
			PRJNA59571	Gi08705	927.68	39.1	22	AERX01	5909	Chromosomes	–
*Oryzias latipes*	Beloniformes; Adrianichthyidae	Japanese rice fish	PRJNA19569	Gi01531	585.33	40.4	–	BAAE01	82496	Scaffolds or contigs	[50]
			PRJNA183868	–	869.82	–	24	BAAF04	7307	Chromosomes	[50]
			PRJNA16702	Gi02165	–	–	–	–	–	–	
*Parabramis pekinensis*	Cypriniformes; Cyprinidae	White Amur bream	PRJNA38199	Gi07171	–	–	–	–	–	–	–
*Paralichthys olivaceus*	Pleuronectiformes; Paralichthyidae	Olive flounder	PRJNA73673	Gi0045242	–	–	–	–	–	–	–
*Pelteobagrus fulvidraco*	Siluriformes; Bagridae	Yellowhead catfish	PRJNA38193	Gi07169	–	–	–	–	–	–	–
*(Tachysurus fulvidraco)*											
*Poecilia formosa*	Cyprinodontiformes; Poeciliidae	Amazon molly	PRJNA89109	Gi0044650	–	–	–	–	–	–	–
*Poecilia latipinna*	Cyprinodontiformes; Poeciliidae	Sailfin molly	PRJNA196862	Gi0048062	–	–	–	–	–	–	–
*Poecilia mexicana*	Cyprinodontiformes; Poeciliidae	Atlantic molly	PRJNA196869	Gi0048063	–	–	–	–	–	–	–
*Psetta maxima*	Pleuronectiformes; Scophthalmidae	Turbot	PRJNA38189	Gi07165	–	–	–	–	–	–	–
*Pundamilia nyererei*	Perciformes; Cichlidae	Python island	PRJNA60367	Gi08441	698.80	40.6	–	AFNX01	7236	Scaffolds or contigs	–
*Rhamphochromis esox*	Perciformes; Cichlidae	–	PRJNA29485	Gi03367	69.87	42.4	–	ABPN01	55751	Scaffolds or contigs	[48]
*Salmo salar*	Salmoniformes; Salmonidae	Atlantic salmon	PRJNA72713	Gi0044519	2435.31	42.6	–	AGKD01	–	Scaffolds or contigs	[103]
*Sebastes nigrocinctus*	Scorpaeniformes; Sebastidae	Tiger rockfish	PRJNA171384	Gi0045199	–	–	–	–	–	–	–
*Sebastes rubrivinctus*	Scorpaeniformes; Sebastidae	Flag rockfish	PRJNA62009	Gi08706	–	–	–	–	–	–	–
*Sparus aurata*	Perciformes; Sparidae	Gilt-head seabream	PRJEA49009	Gi0044643	–	–	–	–	–	–	–
*Stegastes partitus*	Perciformes; Pomacentridae	Bicolour damselfish	PRJNA89147	Gi0044663	–	–	–	–	–	–	–
*Takifugu flavidus*	Tetraodontiformes; Tetraodontidae	Sansaifugu	PRJNA168966	Gi0044522	314.95	45.2	–	AOOT01	34332	Scaffolds or contigs	–
*Takifugu rubripes*	Tetraodontiformes; Tetraodontidae	Pufferfish	PRJNA1434	–	281.57	45.5	22	CAAB02	7091	Chromosomes	[40]
			PRJNA166939	–	391.49	–	–	–	–	–	
			–	Gi18232	–	–	–	–	–	–	
*Tetraodon nigroviridis*	Tetraodontiformes; Tetraodontidae	Green spotted puffer	PRJNA12350	Gc00229	308.45	46.6	–	CAAE01	–	Scaffolds or contigs	–
*Xiphophorus birchmanni*	Cyprinodontiformes; Poeciliidae	Sheepshead swordtail	PRJNA172015	Gi0044901	–	–	–	–	–	–	–
*Xiphophorus clemenciae*	Cyprinodontiformes; Poeciliidae	Yellow swordtail	PRJNA178205	Gi0044902	–	–	–	–	–	–	–
*Xiphophorus hellerii*	Cyprinodontiformes; Poeciliidae	Green swordtail	PRJNA178402	Gi0044903	–	–	–	–	–	–	–
*Xiphophorus maculatus*	Cyprinodontiformes; Poeciliidae	Southern platyfish	PRJNA72525	Gi0045000	652.84	38.8	–	AGAJ01	20640	Scaffolds or contigs	[43]

Adapted from NCBI (http://www.ncbi.nlm.nih.gov/genome/), ENSEMBL (http://www.ensembl.org/index.html) and GOLD (http://www.genomesonline.org/).

Another important resource of fish genomics data is NCBI's Bioproject database (http://www.ncbi.nlm.nih.gov/bioproject), which partially overlaps with the genome database. The Bioprojects database contained almost 900 registered teleost projects (2 July 2013) divided over 12 Project Data Type categories (Table 4). The majority of bioprojects are ‘Transcriptome or gene expression’ projects (84%) and most of the remaining projects are ‘Genome sequencing’ projects (9%). Although the Bioprojects comprise over 168 individual teleost species, only 12 species already account for ∼70% of all projects. Most of the Bioprojects are based on the popular zebrafish model *D. rerio* (37.3%) and other laboratory models, such as fathead minnow (*Pimephales promelas*, 3.4%), mummichog (*Fundulus heteroclitus*, 2.4%), goldfish (*Carassius auratus*, 2.4%), Japanese rice fish/Medaka (*O. latipes*, 1.6%) and three-spined stickleback (*Gasterosteus aculeatus*, 1.3 %). In addition, species that are important for fisheries and aquaculture are well represented, such as rainbow trout (*Oncorhynchus mykiss*, 10%), Atlantic salmon (*Salmo salar*, 5.1%), gilt-head (sea) bream (*Sparus aurata*, 2.2%), Sockeye (red) salmon (*Oncorhynchus nerka*, 1.3%), largemouth bass (*Micropterus salmoides*, 1.3%) and channel catfish (*Ictalurus punctatus*, 1.1%). Also worth mentioning is a set of 30 Bioprojects that include nearly all 28 known species of the genus *Xiphophorus* (swordtails and platyfish), divided over 5 genome and 25 transcriptome projects.

**Table 4:** Teleost Bioprojects registered at NCBI (2 July 2013) according to ‘Project Data Type’

Project Data Type	Number of projects
Transcriptome or gene expression	758
Genome sequencing	80
Epigenomics	21
Refseq genome	12
Variation	8
Map	8
RAD tag	4
Random survey	3
Phenotype or genotype	2
Targeted locus	1
Clone ends	1
Microsatellite	1

Additional draft assemblies of complete teleost genomes have been published, but are not yet available from the NCBI database. For example, genomic scaffolds of the European eel (*Anguilla anguilla*) [7], Japanese eel (*Anguilla japonica*) [6], and the common carp (*C. carpio*) [41] are all accessible via the website www.zfgenomics.com. Recently, a draft assembly of the complete genome of Pacific bluefin tuna (*Thunnus orientalis*) was published [36], which is accessible via GenBank (accession nos. BADN01000001–BADN01133062).

Availability of the complete genome sequence of model and non-model fish species has a strong catalytic effect on a broad range of scientific disciplines and on applied science, as indicated by the following examples. Sequence analysis of the complete genome of the atlantic cod (*Gadus morhua*) uncovered that these cold-adapted teleosts lack a functional major histocompatibility complex (MHC) II pathway. Apparently, this is compensated for by expansion of the number of MHCI genes and by specific adaptations in the Toll-like receptor (TLR) families, thereby providing new fundamental insight into the evolution of the adaptive immune system in vertebrates [39]. The draft genome sequences of the European eel (*A. anguilla*) and Japanese eel (*A. japonica*) showed that these fish species, in contrast to most other teleosts, retained fully populated Hox gene clusters, which may be correlated with their peculiarly complex life cycle that includes two larval stages [6, 7]. In contrast, elasmobranch fishes, such as the cat shark (*Scyliorhinus canicula*) and the little skate (*Leucoraja erinacea*), seem to have lost all HoxC cluster genes [42]. This sheds a completely new light on the relative importance of this family of genes for body plan formation in the fish embryo. Detailed analysis of the genome sequence of the Pacific bluefin tuna (*T. orientalis*) revealed remarkable adaptations in multiple visual pigment genes, which may not only explain their specific predatory behaviour in the blue-pelagic ocean but may also contribute to improved aquaculture conditions [36]. The recent publication of the genome sequence of the platyfish (*Xiphophorus maculatus*) has already significantly broadened our understanding of a wide variety of phenomena, such as live-bearing fish reproduction, pigmentation patterns and melanoma tumorigenesis, and even complex behavioural traits [43].

## CONCLUSIONS AND FUTURE OUTLOOK

The state-of-the-art in genomics of the bony fish has advanced so enormously in the last few years that even in the context of the recent large human sequencing projects, for example in the Encode projects [104], it is no longer possible to catch phrase the recent advances under the term of ‘fishy genomics’ or ‘fish and chips’. The latter catch phrase anyway will have to suffer increasing unpopularity with the prediction that RNA and DNA microarray technologies will soon lose most of their importance, as they will be gradually replaced by methods based on sequencing technologies in the coming years. As explained above, teleost fish species have much to offer for research that is dependent on whole organism test models and for biomedical applications they have in many aspects advantages even over the use of mammalian test systems as recently discussed by Spaink *et al.* [68]. Independently of its applied values, genome-wide studies of the bony fish have great impact for comparative genomics: it will provide a deep understanding of the recent half billion years of evolution in vertebrates and of more recent era that led to an extreme diversification of particular subgroups of the *Teleostei,* such as the cichlids that have been intensively studied from an evolutionary perspective [105]. It will also provide enormous opportunities for data mining and will provide the possibility to trace back the origins of genes from the organisms closest to the earliest evolutionary branches to its origins within invertebrates. For this purpose it is fortunate that many invertebrate species such as the tunicates are also increasingly being analysed with genomics technologies (http://www.tunicate-portal.org/wordpress/). That this can lead to unexpected findings is nicely illustrated by the recent discovery of a completely novel fluorescent protein in the Japanese eel [106]. Furthermore, it can lead to new insights into the origin of individual genes, for instance the interesting example of horizontal gene transfer of a transposon between lamprey species and their hosts indicate that transfer of genetic material between species mediated by parasite–host interactions could be very frequent [107]. In addition to fundamental evolutionary research there will also be important applied aspects, for instance in nature conservation biology and the impact of ancient climate changes on species diversification or extinction processes. This could lead to better prediction models for the effects of current estimated climate changes on biodiversity of the teleost fish species and thereby could provide better guidelines for knowledge-based fishery regulations.

Sequence technology has reached the stage that the capacity of instrumentation is not limiting anymore for sequencing a large number of vertebrates, in contrast to the period at the end of the 20th century when, as an illustration, one of the reasons for sequencing the genome of the Fugu (*Fugu rubripes*) was its small size genome. With the super high capacity of shotgun sequencing facilities it might already now be possible to obtain WGS data for all teleost fish species. Although this would still be extremely costly and no plans have yet been proposed for this, there are bigger problems than cost involved: the bioinformatics and curation facilities that are still not adapted to handle the next-generation sequencing data flow coming from many independent sequencing projects, at least not in a user friendly way. Especially since the quality of WGS shotgun sequences does not make the data highly suitable yet to be integrated in a bioinformatic setting such as ENSEMBL it is needed that complementary bioinformatics and data curation solutions become available at low thresholds to analyse and compare the early versions of WGS assemblies [108]. In addition, it would be desirable to strive to common genome data curation and annotation facilities that cover all fish species as now is offered for zebrafish within VEGA [109] (vega.sanger.ac.uk) and to obtain a comprehensive web site that links all bony fish gene annotations and functional studies following the example presented by ZFIN for zebrafish (zfin.org).

In the context of genome evolution, we can see the great progress in the last years in answering several old questions that have been extensively debated for over decades such as the origin of the *Teleostei* gene duplication. Since it is likely that a majority of all vertebrates will be sequenced within the coming decades, we can get new insights in many fish species into the correlation between genome duplications and repeat content of genomes, on the one hand, with environmental selection pressures and particular adaptations of body architecture. We can also predict that we can soon obtain new insights into the mechanisms that were the cause of gene losses resulting in the trimmed genomes of the modern fishes that we are now studying. This will certainly give an amazing view of the genome dynamics that took place during a period of natural selection that lasted for many hundreds of millions of years. This knowledge can form a bridge between molecular biological studies carried out at the very basic molecular levels in microbes and lower vertebrates and studies in mammalian systems. We have therefore no doubts that genomic studies in the bony fish species will remain to play an important role in uniting the levels of molecular and evolutionary studies, e.g. by being perfect models for system biology studies [60, 61, 110, 111].

**Key Points**

- Next-generation sequencing has revolutionized *de novo* assembly of fish genomes sequences.
- Fish models are rapidly gaining importance at all levels of fundamental and applied science.
- We predict that advances will further accelerate and that the resulting genomic data sets will lead to unprecedented new insights in to vertebrate gene functions and evolutionary mechanisms.
- The application for nucleotide sequencing in transcriptomics technologies will further increase and will gradually replace expression microarray technologies.
- There is an increased need for better and more user-friendly bioinformatic tools and curated database storage of data might become a bottleneck.
